# Astrocytic lipid droplets contain MHCII and may act as cogs in the antigen presentation machinery

**DOI:** 10.1186/s12974-025-03452-0

**Published:** 2025-04-24

**Authors:** Chiara Beretta, Abdulkhalek Dakhel, Khalid Eltom, Fredrik Rosqvist, Simon Uzoni, Tobias Mothes, John S. Fletcher, Ulf Risérus, Dag Sehlin, Jinar Rostami, Wojciech Piotr Michno, Anna Erlandsson

**Affiliations:** 1 Department of Public Health and Caring Sciences, Molecular Geriatrics, Rudbeck Laboratory, Uppsala University, Uppsala, SE-752 37 Sweden; 2 Department of Public Health and Caring Sciences, Clinical Nutrition and Metabolism, BMC, Uppsala University, Uppsala, Sweden; 3 Department of Food Studies, Nutrition and Dietetics, BMC, Uppsala University, Uppsala, SE-751 23 Sweden; 4 Department of Chemistry and Molecular Biology, University of Gothenburg, Gothenburg, 41390 Sweden; 5 Science for Life Laboratory, Uppsala University, Uppsala, SE-752 37 Sweden

**Keywords:** Astrocytes, Lipid droplets, Antigen presentation, Neuroinflammation, Alzheimer’s disease

## Abstract

Lipid droplets (LDs) are crucial for energy homeostasis, but are also involved in a wide spectrum of other cellular processes. Accumulating data identifies LDs as an important player in inflammation. However, the underlying mechanisms and the impact of LDs on neuroinflammation remain unclear. Here, we describe a novel function of LDs in human astrocytes, in the context of Alzheimer’s disease (AD). Although, the overall lipid profile was unchanged in astrocytes with AD pathology, our data show a clear effect on LD metabolism and specific fatty acids involved in neuroinflammation. Importantly, we found astrocytes to be in close contact with infiltrating CD4 + T cells in the AD brain. Moreover, PLIN3 + LDs in astrocytes co-localize with major histocompatibility complex II (MHCII), indicating a role of LDs in adaptive immunity. Comprehensive analysis of human induced pluripotent stem cell (hiPSC)-derived astrocytes revealed that MHCII is in fact loaded within PLIN3 + LDs and forwarded to neighboring cells via tunneling nanotubes and secretion. Notably, the MHCII molecules are cleaved into its active form prior to packing, indicating an alternative route of MHCII shuttling through LDs, transporting functional immune complexes between cells. Quantification of PLIN3 + LDs in astrocytic cultures, human brain tissue and cerebral organoids indicates that AD pathology initially stimulates PLIN3 + LD formation, but in the long-run results in PLIN3 + LD consumption, which may have consequences on the astrocytes’ MHCII distribution capacity. Taken together, our findings present a novel function of PLIN3 + LDs that can be of relevance for AD and other inflammatory conditions.

**Supplementary Information:**

The online version contains supplementary material available at 10.1186/s12974-025-03452-0.

## Background

The human brain is a high-energy consuming organ, which relies on astrocytes for lipid homeostasis [1]. The astrocytes support neurons with energy and store excess fatty acids in lipid droplets (LDs) [2–4]. Recent data emphasize that LDs are not solely lipid storage depots, but complex organelles that can act as hubs of integration between energy metabolism and the immune system [5–8]. By transforming their lipid’s components into active lipid mediators, LDs participate in diverse inflammatory signaling pathways [9]. Moreover, LDs have been shown to be actively involved in antigen cross presentation in dendritic cells [7, 8] and activation of T cells and Natural Killer (NK) cells in the periphery [5, 10, 11]. Importantly, different immune cell responses are connected to modification of the LD profile related to metabolic changes [11]. Due to their key function in inflammatory cell signaling, LDs have been suggested to constitute an attractive candidate for therapeutic intervention [12]. However, the involvement of LDs in brain pathology remains to be investigated.

In neurodegenerative diseases, including Alzheimer’s disease (AD), astrocytes are heavily involved in the inflammatory responses [13]. Thus, unraveling the immune regulatory function of astrocytic LDs will give important insight into underlying disease mechanisms and possibly lead to identification of new therapeutic targets. Although LDs can be found in all species from yeast to man, their number, size and composition varies widely between cells or even within the same cell [14, 15]. Different subgroups of LDs have been identified, based on their perilipin (PLIN) coating proteins [16, 17], but their functional difference in health and disease remain unknown. In astrocytes, PLIN3 + LDs are the most prominent LD population [18], making this subgroup extra interesting in neuroinflammation. Recent reports indicate that astrocytes indeed act as antigen presenting cells, both in vitro and in the human brain [19–21]. However, the link between lipid handling and the astrocytes’ antigen presenting capacity has not been investigated previously. Here, we report for the first time that LDs represent a distinct antigen presentation machinery in astrocytes, acting as modulators and transport packages for MHCII proteins.

## Materials and methods

### Immunohistochemistry

#### Deparaffinization

Brain sections from cortex of AD patients and age matched controls were used (kindly provided from Uppsala Biobank by Professor Irina Alafuzoff, ethical number 2012/494). The thickness of the analyzed sections was 5 μm. To ensure that exactly the same region was analyzed from all patients and controls, a cutting mold was used during the sectioning procedure. Prior to immunostaining, the sections were deparaffinized using xylene 2 × 10 min, rehydrated using 99.5% ethanol 2 × 10 min, 95% ethanol for 10 min, and 70% ethanol for 10 min and washed in PBS 3 × 5 min.

#### Blocking

The samples were blocked for 30 min with 5% NGS in 0.1% saponin in PBS.

#### Primary antibody incubation

Primary antibodies (Table 1) were diluted in 0.1% saponin, 0.5% NGS in PBS incubated with the sections at 4 °C overnight. The following day, the sections were washed in PBS 3 × 5 min.

#### Secondary antibody incubation

Secondary antibodies were diluted in 0.1% saponin, 0.5% NGS in PBS. The secondary antibodies used were AlexaFluor 488, 555 or 647 against mouse, rabbit, rat or chicken (1:200, Molecular Probes). The samples were incubated with the secondary antibodies for 1 h at 37 °C, washed in PBS 3 × 5 min and then dipped in 70% ethanol five times, incubated in 0.3% Sudan black for 20 min, dipped in 70% ethanol twelve times, washed in PBS again, and mounted using EverBrite Hardset mounting medium with DAPI (Bionordika).

### Proximity ligation assay

PLA was performed using the DuoLink insitu Brightfield kit (DUO92012, Sigma Aldrich), according to the manufacturer’s instructions. In short, human brain tissue sections were deparaffinzed as described above, washed in PBS for 10 min and incubated with DuoLink peroxidase solution for 30 min, to block endogenous peroxidase activity. After washing 2 × 5 min in TBS-T, the tissue were blocked and permeabilized using DuoLink blocking solution for 1 h at 37 °C. Primary antibodies for PLIN3 and MHCII (Table 1) were diluted in DuoLink Antibody Diluent and added to the sections, to be incubated at 4 °C overnight. The sections were then washed 2 × 5 min with TBS-T and incubated with the DuoLink PLA probes (anti-mouse MINUS and anti-rabbit PLUS) in DuoLink Antibody Diluent for 1 h at 37 °C. After 2 x washes in TBS-T, a ligation step was performed with ligase diluted in DuoLink Ligation buffer for 30 min at 37 °C, in order to yield the hybridized circular oligonucleotide. Following 2 x washes amplification was performed in polymerase in DuoLink Amplification buffer for 2 h at 37 °C. The sections were then washed an incubated with DuoLink Brightfield Detection buffer for 1 h at RT. After another washing step, substrate development was carried out by adding substrate A, B, C, and D for 2 min. The sections were counterstained with Mayer’s Hematoxylin solution for 1 min, followed by washing in running dH2O for 10 min and finally re-hydrated and mounted with Pertex.

**Table 1:** Primary antibodies used in this study

Antibody	Host	Technique	Dilution	Company	Cat #
Vimentin	Chicken	ICC	1:200	Sigma Aldrich	AB5733
GLAST-1	Rabbit	ICC	1:200	Novus Biologicals	NB100-1869
AQP4	Rabbit	ICC	1:200	Novus Biologicals	NBP1-87679
GFAP	Chicken	ICC, IHC	1:200	Abcam	ab4674
PLIN1	Rabbit	ICC	1:100	Abcam	ab3526
PLIN2	Rabbit	ICC	1:100	Abcam	ab52356
PLIN3	Rabbit	ICC, TEM, IHC	1:100	Abcam	ab47638
MHC-II	Mouse	ICC, TEM, IHC	1:100	DAKO	M0775
MHCII clone L432	Mouse	ICC	1:100	Proteintech	65218-1
S100β	Mouse	ICC	1:200	Sigma Aldrich	S2532
LAMP1	Rabbit	ICC	1:100	Abcam	ab24170
CD74	Rabbit	ICC	1:200	Invitrogen	PA522113
CD4	Rabbit	IHC	1:200	Abcam	ab133616
CD4	Mouse	IHC	1:200	Invitrogen	MA1-7631

### Culture of human iPSC derived astrocytes

Human induced pluripotent stem cell (iPSC)-derived neuroepithelial stem (NES) cells (iPSCs, Cntrl9 II cell line) were differentiated into astrocytes following a well-established 28-day protocol [22]. Immunostainings, using the astrocyte marker S100β and the actin marker phalloidin, demonstrated that all cells in the culture were astrocytes (Fig S1). Astrocyte differentiation medium consisted of advanced DMEM/F12 (ThermoFisher, 12634-010) supplemented with 1% Penicillin Streptomycin (ThermoFisher, 15140-122), 1% B27 supplement (ThermoFisher, 17504-044), 1% non-essential amino acids (Merc Millipore) and 1% L-Glutamine (ThermoFisher, 25030-024). The following factors were added to the medium just before use: basic fibroblast growth factor (bFGF) 10 ng/ml (ThermoFisher, 13256029), heregulin β-1 10 ng/ml (Sigma-Aldrich, SRP3055), activin A 10 ng/ml (Peprotech, 120-14E) and insulin-like growth factor 1 (IGF-1) 200 ng/ml (Sigma-Aldrich, SRP3069). From day 15, 20 ng/ml ciliary neurotrophic factor (CNTF; ThermoFisher, PHC7015) was also included. Culture medium was changed every other day until day 28. For experiments, fully differentiated astrocytes were detached using 4% trypsin-EDTA (Thermo Scientific, 10779413) and seeded at 5 000 cells/cm^2^ on pre-coated plates.

### Production of amyloid Β soluble aggregates

Aβ_42_ soluble aggregates were produced according to a well-established protocol [23, 24]. In short, synthetic Aβ_42_ peptides (Innovagen, SP-BA42-1) were dissolved in 10 mM NaOH, mixed with phosphate-buffered saline (PBS) to a concentration of 443 μM (2 mg/ml) and incubated at 37 °C for 30 min. HiLyte™ Fluor 555-labeled soluble aggregates were produced using the same protocol, by mixing unlabeled Aβ_42_ 1:1 with HiLyte™ Fluor 555-labeled Aβ_42_ (Anaspec, AS-60480-01). The samples were centrifuged for 5 min at 17 900 x g at 4 °C to remove any insoluble aggregates, followed by 1:4 dilution of supernatant in sterile PBS to a final concentration of 0.5 mg/ml. The protofibril specific ELISA, based on mAb158 [23], was used to confirm the Aβ_42_ soluble aggregate concentration and transmission electron microscopy (TEM) was used to verify their shape. For the TEM analysis, Aβ soluble aggregates were diluted 1:10 in MilliQ H_2_O and dropped onto carbon coated 300-mesh copper grids, negatively stained with 1% Uranyl acetate for 5 min and air dried. The samples were then analyzed using a Hitachi H-7100 transmission electron microscope. A careful characterization of the Aβ_42_ soluble aggregates has been performed previously, using HPLC-SEC, Thioflavin T staining, and different ELISAs, in addition to TEM [23–25].

### Aβ_42_ soluble aggregate exposure

Astrocytes were exposed to 0.2 μM Aβ_42_ soluble aggregates in astrocyte medium for 7 days. Parallel control cultures received medium without Aβ. After three days of Aβ exposure an extra half volume of fresh medium was added, without removing any conditioned medium. At day 7 the cells were washed 3 times with medium and were either fixed/lysed (day 7d) or maintained in Aβ-free medium for another 6 days with addition of an extra half volume of fresh medium after 3 days (day 7d + 6d). Conditioned medium was collected at 7d and 7d + 6d.

### Oleic acid exposure

Astrocytes were exposed to 20μM oleic acid (Sigma Aldrich, O1008-1 g) for 7 days. Parallel control cultures received medium without oleic acid. After three days of exposure an extra half volume of fresh medium with oleic acid was added, without removing any conditioned medium.

### Immunocytochemistry

Cells were fixed with 4% paraformaldehyde (PFA) (Sigma-Aldrich, P6148) in PBS for 15 min at RT, washed 3 times with PBS and permeabilized and blocked with 0.1% saponin, 5% normal goat serum (NGS) (Bionordika, S-1000) in PBS for 30 min at RT. The cells were then incubated with primary antibodies (Table 1) for 1 to 4 h at RT, washed 3 times with PBS and incubated with secondary antibodies for 45 min at 37 °C. All antibodies were diluted in 0.1% saponin, 0.5% NGS in PBS. The secondary antibodies used were AlexaFluor 488, 555 or 647 against mouse, rabbit, rat or chicken (1:200, Molecular Probes). Next, the cells were washed 3 times with PBS and mounted on microscope slides using EverBrite hard-set medium with or without DAPI (230032, Biotium). Images were captured using a fluorescence microscope Observer, Z1 Zeiss, using 40x or 63x objectives, and a confocal Zeiss LSM700, using 63x objective.

#### Dyes

To visualize actin, cells were incubated 30 min in phalloidin-fluorescein dye at RT (1:1000, Abcam, ab176759). To visualize neutral lipids, cells were incubated with BODIPY 493/503 (1:100, ThermoFisher, D3922) or LipidTOX (1:50, Invitrogen, H34476) for 30 min at 37 °C.

### Lipid droplet isolation

Cells were detached using 4% trypsin-EDTA (Thermo Scientific, 10779413), centrifuged at 300xg for 5 min, washed with PBS, and centrifuged again. Lipid droplets were isolated from pellets consisting of 2 million cells using the Lipid Droplet Isolation Kit (ab242290, Abcam).

### Transmission electron microscopy

#### Negative staining of lipid droplets

5 μl of isolated lipid droplets were placed on a formvar- and carbon coated 200-mesh copper grid (Ted Pella), let dry and then additional 5 μl of the sample were added to the same grid. The excess solution was removed with filter paper. The grid was subsequently washed two times by placing the grid on drops of MQ water directly followed by contrasting two times on a drop with 2% uranyl acetate. Excess of uranyl acetate was removed by filter paper and the grid was air dried.

#### Immunogold labelling

A volume of 5 μl of isolated lipid droplets was placed on a formvar- and carbon coated 200-mesh nickel grid (Ted Pella). The excess solution was removed with filter paper. The grid was subsequently washed two times on drops of MQ water directly followed by blocking solution for goat conjugates (Aurion, Wageningen) for 15 min at RT. After wash with BSA-C™ (0.1%) (Aurion, Wageningen), the grids were placed on a drop of primary antibodies diluted in BSA-C™ for 40 min (Table 1). The grids were washed in BSA-C™ and then incubated with a goat anti-mouse secondary antibody conjugated with a 12 nm gold particle (Jackson ImmunoResearch) mixed with a goat anti-rabbit secondary antibody conjugated with a 6 nm gold particle (Jackson ImmunoResearch) for 40 min. After extensive washing in BSA-C ™ and MQ water, the grids were contrasted with 2% uranyl acetate twice. Excess of uranyl acetate was removed with filter paper and the grid was air dried.

### Time-lapse microscopy

Time-lapse experiments were performed at 37 °C in humidified 5% CO2 in air, using a Nikon Biostation IM Live Cell Recorder (Nikon). The cells were cultured at a concentration of 5000 cells/cm2 in time-lapse culture dishes (VWR) and images were captured every 10 min for 48 h for the transfer analysis. Movies of the time points of interest were exported at a speed of 4 frames/second.

### Lipid droplet transfer experiments

#### Donor-acceptor co-culture set up

The cultures used for lipid droplet transfer experiments were all from the 7d + 6d time point. Prior to co-culturing, the donor cultures (2500 cells/ cm^2^) were incubated with LipidTOX (1:100) for 2 h, and the acceptor cultures (2500 cells/ cm^2^) were stained with BioTracker 655 Red Cytoplasmic Membrane Dye (SCT108, Sigma-Aldrich) following manufacturer’s instructions. After incubation, both donors and acceptors were washed and the acceptor astrocytes were trypsinized and added to the donor cultures. After 18 h co-culturing the astrocytes were fixed with 4% PFA as described above, and mounted using EverBrite Hardset mounting medium with DAPI (Bionordika).

#### Donor-acceptor conditioned medium set up

Donor astrocytes were incubated with LipidTOX (1:100) for 2 h, washed and cultured with low volume of medium (55 μl/cm^2^) for 18 h. Conditioned medium was collected and added to acceptor astrocytes for 6 h prior to fixation and mounting using EverBrite Hardset mounting medium with DAPI (Bionordika) and Phalloidin (1:100).

### Lipid extraction and profiling

Cells were detached using 4% trypsin-EDTA (Thermo Scientific, 10779413), centrifuged at 300xg for 5 min, washed with PBS, centrifuged again as before. Pellet was extracted using an adapted MTBE protocol [26]. Briefly, 100 μL of cold MeOH was added to the pellet, samples were sonicated for 1 min in an ice bath, and then vortexed for 1 min. Subsequently, 400 μL of cold MTBE was added to the sample, and the samples were vortexed for 5 min. Finally, 100 μL of ddH2O was added to the samples. Samples were centrifuged at 10,000xg for 10 min at 4 °C, and the upper organic phase was collected and dried using a vacuum-centrifuge. Samples were reconstituted in 90% MeOH: ddH2O. MALDI-TOF analysis was performed through dry droplet method, on a 384-well ground steel MALDI target plate, using 10 mg/mL α-Cyano-4-hydroxycinnamic acid (CHCA) in 70% MeOH: ddH2O as the matrix on a timsTOF (Bruker) instrument. Overview spectra from two control samples and two treated samples were compared.

### Time of flight secondary ion mass spectrometry (ToF-SIMS)

ToF-SIMS analysis was conducted using an Ionoptika J105 *3D Chemical imager* (Ionoptika Ltd, UK), described in *Fletcher et al. (2008)* [27]. The instrument is equipped with a 40 kV gas cluster ion beam using (CO_2_)_6000_^+^ as the primary ion beam. Every sample was analyzed in four different locations, two in each polarity, producing mass spectral images covering 1200 × 1200 μm2 areas with a 256 × 256 pixel image acquired at each location. The recorded mass range was mass-to-charge ratio (m/z) 100–2000. The samples were pre-etched with an ion dose of 3.41 × 10^12^ ions/cm^2^ to remove the cell membrane and expose the lipid droplets, and the interior of the cells was analyzed with a combined ion dose of 1.02 × 10^13^ ions/cm^2^.

### Lipid extraction for fatty acid profiling

Cells were detached using 4% trypsin-EDTA (Thermo Scientific, 10779413), centrifuged at 300xg for 5 min, washed with PBS, centrifuged again as before. The pellet of 1.8 million cells was then resuspended in 500 μl PBS. The cell samples were mixed with 5 ml chloroform/methanol (2:1 v/v) to extract total lipids, followed by the addition of 1 ml of 1 M NaCl. The solvent phase was collected and evaporated to dryness using nitrogen gas. Cholesteryl esters (CE), triacylglycerols (TG), non-esterified fatty acids (NEFA) and phospholipids (PL) were separated using solid-phase extraction according to Burdge et al. [28], with slight modifications. Fatty acid methyl esters were prepared using sulphuric acid in methanol (6% v/v). Fatty acid composition in the respective fractions were analyzed using an Agilent GC 7890B with a fused silica capillary column (Supelco SP-2380, 100 m x 0.25 mm x 0.20 μm). Peaks were identified by comparison to a known reference standard (Supelco 37 component FAME mix). Fatty acids are presented as the relative sum of all fatty acids analyzed (area%).

### Culturing of human iPSC derived cortical organoids

Human cortical organoids were generated from induced pluripotent stem cells (iPSC, CNTRL9 II cell line). The hiPS cells were cultured on 6-well cell-culture plates (Thermo, 140675) coated with 50 μg/mL vitronectin (Thermo, A14700), using E8 + medium (Thermo, A1517001). The medium was replaced every day and the cells were passaged at 80–90% confluency, using accutase (Thermo, 11599686). The cells were resuspended in E8 + medium fortified with 1x Y27 (Cayman, Caym10005583-10) and transferred as single cells to an AggreWell (Stemcell, 34411) (3 M cells/well). The AggreWell plate was centrifuged at 300 g for 5 min and placed in an incubator over night to create spheroids. The spheroids were then gently collected and sieved through a cell strainer (Fisher Scientific, 10737821). The spheroids were kept on UltraLow adhesion 100 mm plates (Thermo, 16855831) in E6 + medium (Thermo, A1516401) fortified with 10 μM SB431542 (Cayman, Caym13031-10), 0,25 μM XAV939 (Cayman, Caym13596-10) and 2.5 μM Dorsomorphine hydrochloride (Cayman, Caym21207-25). From the second day, the medium was changed every day. After 5 days the medium was substituted for Neurobasal (Thermo, 21103049) containing 1% penicillin-streptomycin (Thermo Fisher, 11548876), 1x B27-noVitA (Fisher Scientific, 11500446) and 1x GlutaMAX (Thermo Fisher, 35050038). For the first two weeks in Neurobasal, the medium was replaced every day, but for the coming four weeks it was replaced every other. From day 6–24, 27 ng/mL FGF2 (Fisher Scientific, 10222253) and 20 ng/mL EGF (Fisher Scientific, 17159651) were added to the medium. From day 25–43, 20 ng/mL NT-3 (Fisher Scientific, 17109511) and 20 ng/mL BDNF (Fisher Scientific, 17169321) were added to the medium. From day 44 and onwards, the medium had no additional factors and it was changed twice a week.

### Exposure of organoids to protein aggregates and astrocytes with protein inclusions

10 weeks old organoids were transferred to individual wells of a 96-well UltraLow adhesion plate (Fisher Scientific, 174932) containing 100 μL of medium. Then, another 100 μL of medium was added with or without 0.2 μM Aβ_42_ soluble aggregates. The organoids were cultured separately and the medium replaced every other day. After one week, the organoids were transferred to culture dishes and cultured for further 10 weeks with regular medium changes. At 10 weeks the organoids were fixed or lysed. They were fixed overnight in 4% PFA. The next day, they were transferred to 30% sucrose solution until sectioning. Organoids were snap-frozen on dry ice and sectioned as 10 μm thick slices using a Leica CM1860 UV cryostat and placed on coverslips. The coverslips were set to dry over night at RT and subsequently stored in -20 °C until analysis.

### Data analysis

#### IHC

All data presented is based on analysis of cortical and hippocampal brain tissue sections from 10 AD patients and 10 age matched controls. The tissue was stained for PLIN3 and GFAP and 15 pictures/individual were captured for the grey respective white matter of cortex (Fig. S2b) and for the CA4 region of hippocampus (Fig. S2c). The number and area of lipid droplets (LDs) were analyzed using a custom ImageJ macro, which quantified only LDs that met predefined criteria for diameter and circularity consistent with known LD characteristics (Table S1-2).

#### ICC

All data presented is based on three independent cell culture experiments. For the quantification of MHCII and MHCII + LDs 220–550 pictures were taken with 40x objective and quantified by using custom-made ImageJ macro (Table S3). For the quantification of donor acceptor experiments 15 pictures were captured with 40x objective. The number of LDs was manually assessed and the astrocytes were divided in three groups: astrocytes with no LDs, low number of LDs (1–3 LDs/cell) or high number of LDs (> 3 LDs/cell). The astrocytes were stained for PLIN 3 and 15 pictures were captured as 12-image Z-stacks for each replicate with 40x objective. The Z-stack were then compiled in composites showing the sum of the PLIN3 signal with custom made macro (Table S4).

#### ToF SIMS

Two control samples and three treated samples were analyzed.

From the images recorded in negative ion mode, the number of collected mass spectra was 22 in control sample 1, 28 in control sample 3, 21 in Aβ sample 1, 45 in Aβ sample 2, and 29 in Aβ sample 3. A total of 50 mass spectra were recorded from the control samples and 97 from the Aβ samples. Using Ionoptika Image Analyser 3.3.10 (Ionoptika Ltd, UK), multiple mass spectra were collected in every image from areas with high intensity of fatty acids (m/z 253.2 (FA 16:1), 255.2 (FA 16:0), 281.2 (FA 18:1), 283.2 (FA 18:0), and 303.2 (FA20:4) in negative ion mode. The mass spectrum of every cell was extracted from a 7 × 7 pixel area in the images to Microsoft Excel (Microsoft Corporation, WA, USA).

The Excel file containing the mass spectra were imported to MATLAB R2022b (9.13.0.2166757 Update 4) (MathWorks Inc., US-MA) and ChiToolbox [29] was used to peak pick and centroid the data. The peak picked mass spectra were normalized and analyzed with principal components analysis (PCA). The LIPID MAPS Structure Database (LMSD) [30] was used for putative lipid identification.

### Statistical analysis

The Shapiro-Wilks normality test excluded normal data distribution. Therefore, the data was analyzed by nonparametric Mann Whitney test or Kruskal Wallis test with Dunn’s multiple comparison, using GraphPad Prism (version 5.03) and presented in the appropriate graph (details shown in the figure legend for each graph). The level of significance for all the graphs is: * = *P* < 0.05, ** = *P* < 0.01, and *** = *P* < 0.001.

Principal component analysis (PCA) was used to analyze the data from ToF-SIMS analysis.

## Results

### Astrocytes interact with CD4 + T cells

**Fig. 1:**
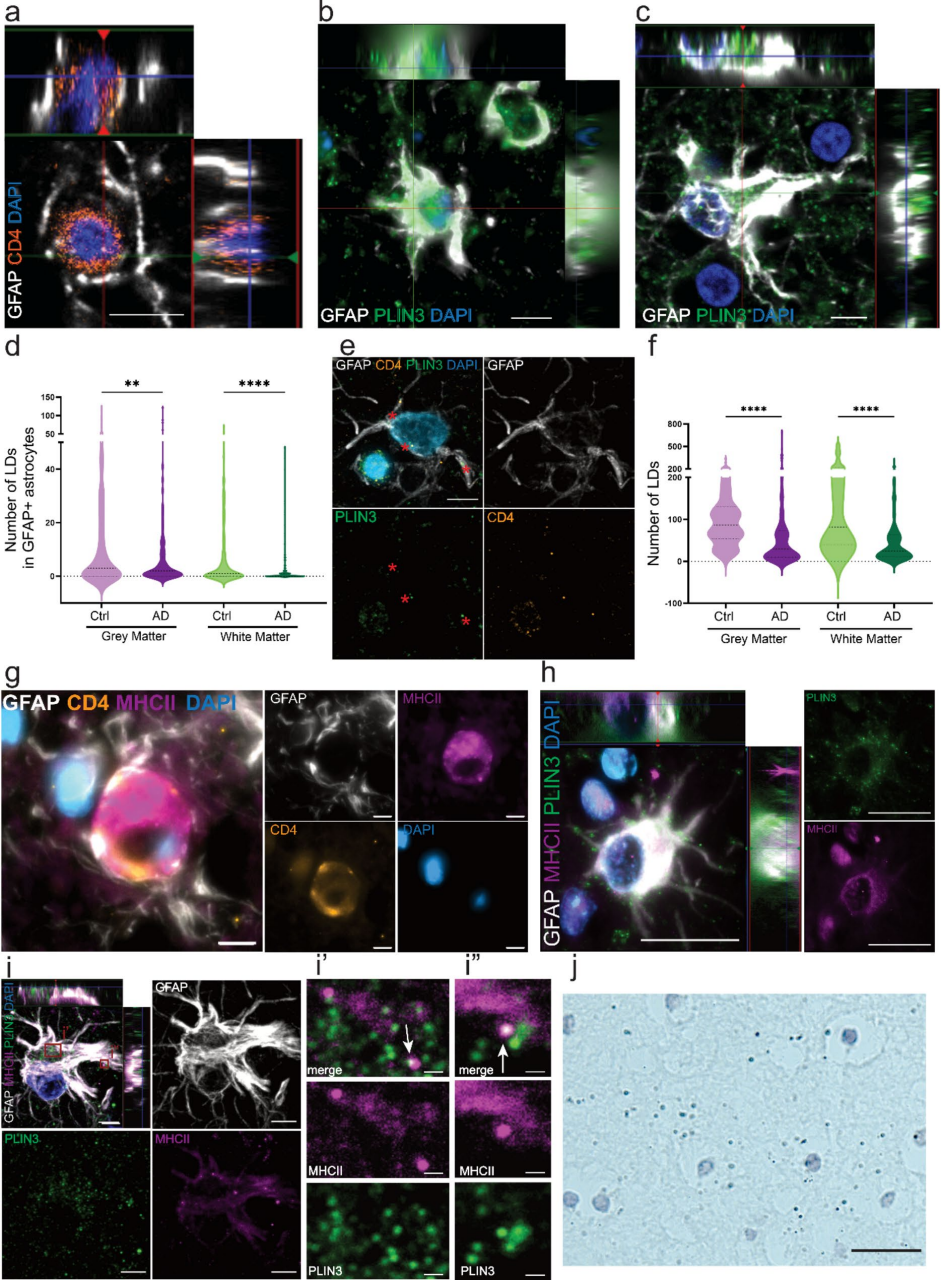
Astrocytes contain MHCII + LDs and interact with CD4 + T cells in the human brain. IHC demonstrate that astrocytes are in close contact with CD4 + T cells in the human brain (**a**). A proportion of the GFAP + astrocytes have a very high PLIN3 + LD load, both in cortex (**b**) and hippocampus (**c**). Quantification of PLIN3 + LDs in astrocytes revealed a significant decrease in AD brains, compared to control brains (**d**). T cells are in direct contact with astrocytes containing PLIN3 + LDs (stars) (**e**). The total number of PLIN3 + LDs is also significantly lower in AD brains, compared to control brains (**f**). For the statistical analysis of (**d**) and (**f**) the N number is 10 ctrl and 10 AD patients and was shown as violin plots with median and interquartile ranges. The level of significance for all the graphs is: * = *P* < 0.05, ** = *P* < 0.01, and *** = *P* < 0.001. MHCII + astrocytes are in direct contact with CD4 + T cells (**g**). PLIN3 + LDs are present in MHCII + astrocytes in cortex (**h**) and in hippocampus (**i**). Higher magnification confirmed co-localization of PLIN3 + LDs and MHCII (white arrows,** i’-i”**). PLA of PLIN3 and MHCII showed positive puncta, verifying direct contact between the proteins in the human brain (**j**). Scale bar (**a**,**c**,**e**, **g**,**i**): 5 μm; (**b**):10 μm; (**h**, **j**): 20 μm; (**i’,i”**): 1 μm

Neuroinflammation is a major pathological hallmark of AD, but the underlying mechanisms remain poorly understood. In a previous study, we quantified the number of MHCII + astrocytes and MHCII + microglia in the Parkinson’s disease brain. Our results showed that many astrocytes express MHCII and may be highly responsible for T-cell activation [21]. By analyzing human tissue sections, we here demonstrated a direct interaction between GFAP + astrocytes and CD4 + T cells in the AD brain (Fig. 1a, Fig. S2a). In the periphery, LDs have been shown to play a critical role in T cell activation [5–8, 10]. Hence, we sought to investigate the link between LDs and antigen presentation in astrocytes. In consistence with previous reports [18], we found perilipin 3 (PLIN3) coated LDs to be situated inside GFAP + astrocytes, in both cerebral cortex and hippocampus (Fig. 1b-c). Quantification of astrocytic PLIN3 + LDs in cortical grey and white matter (Fig. S2b) showed a significant decrease in AD, compared to age matched controls (Fig. 1d). However, there was no significant difference between the groups in the CA4 region of hippocampus (Fig. S2c-d), indicating that PLIN3 + LDs in astrocytes are affected differently across brain regions. Moreover, T cells in close contact with astrocytes were notably rich in PLIN3 + LDs (Fig. 1e, Fig. S2e), suggesting a possible LD interplay between infiltrated T cells and astrocytes in the AD brain. Quantification of the overall PLIN3 + LD content in AD brain tissue, revealed a significant decrease in both the number (Fig. 1f) and the surface area of LDs (Fig. S2f) in cortex, while the levels were unchanged in hippocampus (Fig. S2g-h). Imaging of astrocytes in contact with CD4 + T cells in the human brain further confirmed that astrocytes were positive for MHCII in the contact zone with T cells (Fig. 1g). Interestingly, we noted that astrocytic PLIN3 + LDs co-localized with MHCII both in cortex (Fig. 1h) and in hippocampus (Fig. 1i, Fig. S2i-j). To verify the connection between MHCII and PLIN3 in the human brain, we used proximity ligation assay (PLA), which will give rise to a positive signal only if the proteins are at a distance of 40 nm or less, therefore implying protein-protein interactions between the two proteins of interest [31]. PLA analysis, using PLIN3 and MHCII antibodies, showed distinct puncta in the AD brain, confirming a direct interaction between the two proteins (Fig. 1j, Fig. S3), indicating a new role for LDs in astrocyte-mediated neuroinflammation.

### Human astrocytes store MHCII molecules within LDs

**Fig. 2:**
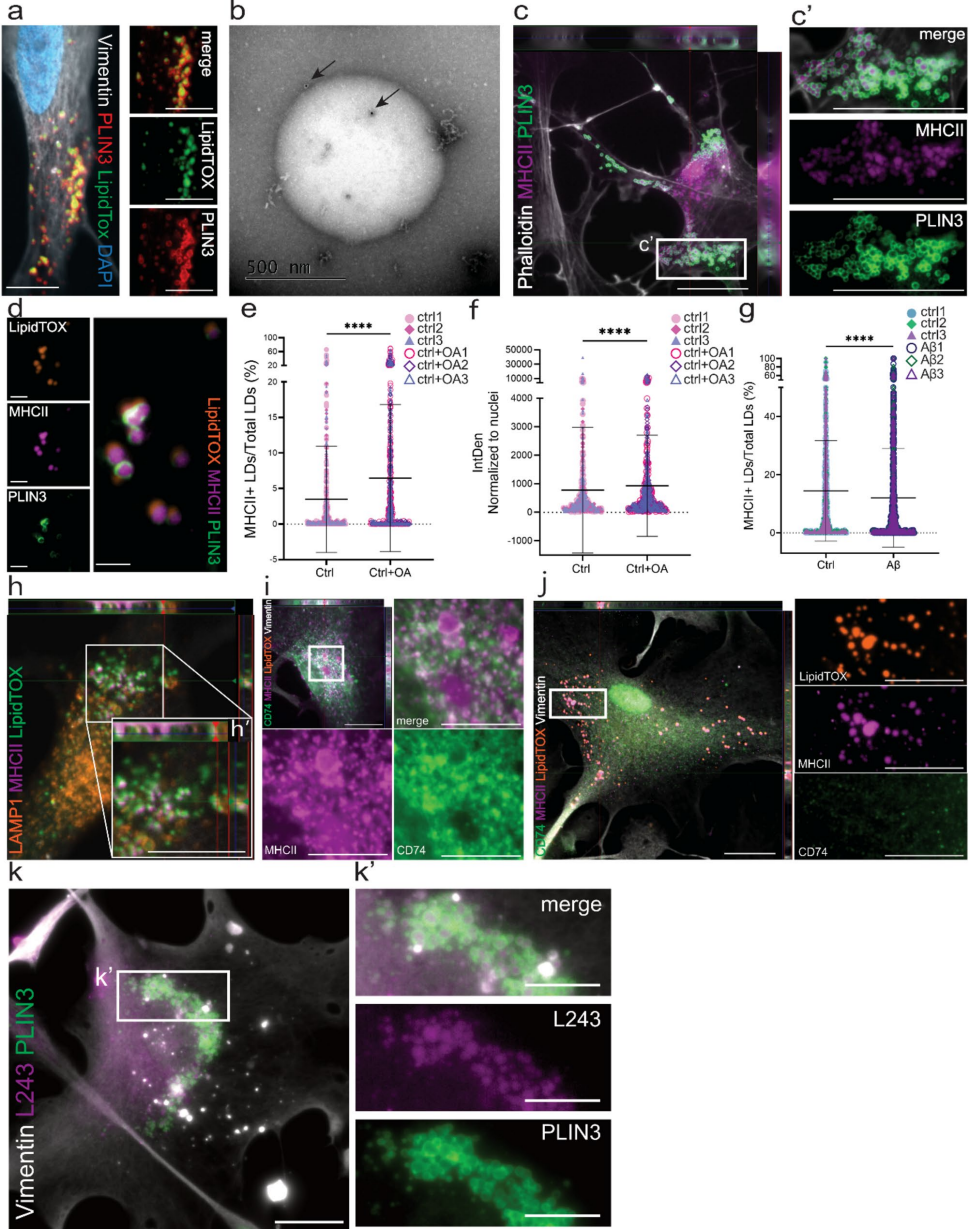
MHCII is stored within astrocytic LDs. ICC of PLIN3 in combination with LipidTOX, show distinct PLIN3-coated LDs in hiPSC-derived astrocytes (**a**). ImmunoTEM of isolated LD verifying the presence of PLIN3 (arrows) on the surface of LDs (**b**). In some astrocytes, MHCII is situated within PLIN3 coated droplets (**c**). LipidTOX staining confirming MHCII co-localization with LDs (**d**). Exposure to OA resulted in a significant increase of both the percentage MHCII + LDs (**e**) and total MHCII levels (**f**), compared to control astrocytes. The percentage of MHCII + LDs, showed a significant decrease in Aβ exposed astrocytes compared to control astrocytes (**g**). For the statistical analysis of **e-g** the N number is 3 (shown in the graphs with different symbols and colors). Data is shown as scatterplots with mean and standard deviation. The level of significance for all the graphs is: * = *P* < 0.05, ** = *P* < 0.01, and *** = *P* < 0.001. LDs and MHCII are not situated in LAMP1 + endosomal/lysosomal vesicles (**h**). CD74 co-localizes with cytoplasmic MHCII (**i**), but not with MHCII situated in LDs (**j**). MHCII signal detected with the active conformation specific clone L432 within PLIN3 coated droplets (**k**). Scale bar (**a**-**c**, **h**-**j**, **k**): 20 μm; (**d**): 2 μm; (zoomed in **i**-**j**, **k**’): 10 μm

To further examine the involvement of LDs in astrocytic antigen presentation, we performed experiments using hiPSC-derived astrocytes, expressing the cell specific markers GLAST, GFAP, AQP4, S100β, and vimentin (Fig. S4a-e). ICC analysis of the astrocytes demonstrated that neither PLIN1, nor PLIN2 co-localized with BODIPY 493/503 and LipidTOX labelled LDs (Fig. S4f), while PLIN3 clearly coated the astrocytic LDs (Fig. 2a, Fig. S4g). In addition, immunoTEM analysis of isolated LDs confirmed PLIN3 expression on the LD surface (Fig. 2b). Notably, ICC showed that MHCII was situated in the core of PLIN3 + LDs, in some astrocytes (Fig. 2c-c’). This striking finding was verified by LipidTOX/MHCII/PLIN3 triple stainings (Fig. 2d). Importantly, addition of unsaturated fatty acid oleic acid (OA) (known to cause LD accumulation) demonstrated that LD buildup in astrocytes increased the percentage of MHCII + LDs (Fig. 2e), as well as the overall MHCII expression (Fig. 2f), suggesting that LD accumulation promotes antigen presentation. To investigate if the MHCII-loaded LDs were affected by AD pathology, we next exposed astrocytes to amyloid β (Aβ) soluble aggregates for 7 days (Fig. S4h-i). Quantification of the percentage of MHCII + LDs out of total LDs, showed a significant decrease in Aβ exposed astrocytes, compared to control astrocytes (Fig. 2g), which corresponded well to the general drop of MHCII expression in the Aβ astrocytes (Fig. S4j).

MHCII is known to be processed within the endo-lysosomal pathway and is shuttled to and from the cellular membrane in LAMP1 positive vesicles [32]. However, ICC revealed no co-localization between LAMP1 and the MHCII + LDs (Fig. 2h), indicating that LDs with MHCII cores were not situated in endo-lysosomal vesicles. We reasoned that the MHCII molecules that are packed into LDs may already have gone through endo-lysosomal processing. Initially, MHCII contains an invariant chain (Ii), which is cleaved off in the lysosome, to allow MHCII antigen binding [32]. Ii cleavage results in the release of CLIP and CD74. The latter is then transported to the cell membrane where it acts as a cytokine receptor [32]. ICC showed that when MHCII was evenly expressed in the cytoplasm, it co-localized with CD74 (Fig. 2i). However, when MHCII was present within LDs, it did not (Fig. 2j), suggesting that MHCII is loaded within LDs after the endo-lysosomal processing. To confirm this finding, we performed ICC with the L432 clone antibody that detects a functional MHCII conformation. Importantly, the result shows that MHCII situated in the astrocytic LDs is indeed functional (Fig. 2k). Taken together, our data strongly indicates involvement of LDs in astrocytic antigen presentation and identify a novel route of intracellular MHCII shuttling.

### Astrocytes transfer LDs to neighboring cells via TNTs and secretion

**Fig. 3:**
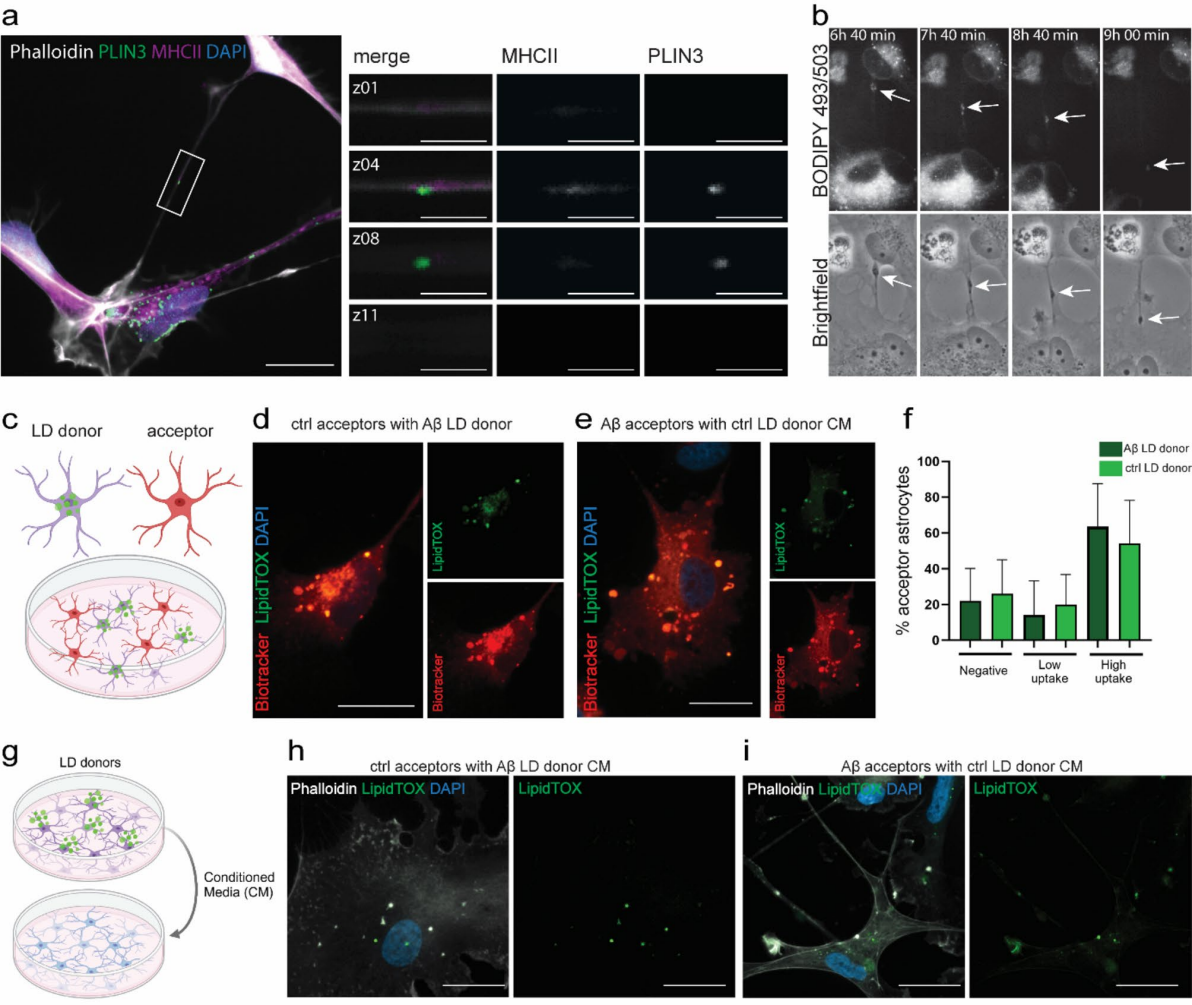
Lipid droplets are transferred between astrocytes via TNTs and secretion. 3D z-stack image of a PLIN3 + LD and MHCII co-localizing within a TNT (**a**). Time-lapse microscopy follow transfer of LDs through TNTs over time (white arrow, **b**). Schematic illustration of the LD donor-acceptor experimental set-up (**c**). Cell-to-cell transfer of LDs occur both from Aβ-exposed astrocytes to control astrocytes (**d**) and from control astrocytes to Aβ-exposed astrocytes (**e**). Quantification of acceptor astrocytes with no detectable LDs (negative), low number of LDs (1–3 LDs/cell), or high number of LDs (> 3 LDs/cell) (**f**). For the statistical analysis of (**f**) N number is 3 and the data is presented in a bar graph with standard deviation. The level of significance for all the graphs is: * = *P* < 0.05, ** = *P* < 0.01, and *** = *P* < 0.001. Schematic illustration of the conditioned medium experiments (**g**). Lipid droplets were secreted and taken up by acceptor cells, both when Aβ exposed astrocytes (**h**) and control astrocytes (**i**) were donors. Scale bar (**a**, **d**,**e**, **h**,**i**): 20 μm; (zoomed in **a**): 5 μm

We have previously shown that aggregated proteins, as well as MHCII molecules and mitochondria can be transported from one astrocyte to another via tunneling nanotubes (TNTs) [21, 33–35]. Here, we sought to explore whether LDs are transferred between astrocytes in a similar way. Indeed, ICC demonstrated that human astrocytes transfer both LDs and MHCII between each other via TNTs (Fig. 3a). Using time lapse microscopy, we were able to follow the transfer of LDs between astrocytes, exposed to BODIPY493/503, through TNTs (Fig. 3b, Movie S1). To investigate the extent of LD transfer we performed two sets of donor-acceptor experiments, in which one astrocyte population was pre-labelled with LipidTOX staining their LD content (LD donors) and another astrocyte population was pre-labelled with Biotracker cytoplasmic Membrane dye, which stained the membranes of the cells (acceptors) (Fig. 3c). In the first set-up, Aβ-exposed astrocytes were LD donors and control astrocytes acceptors and in the second set-up, control astrocytes were LD donors and Aβ-exposed astrocytes acceptors. Fluorescent imaging showed that LipidTOX positive LDs were transferred to the acceptors, both when the donors were Aβ-exposed astrocytes (Fig. 3d) and control astrocytes (Fig. 3e). For quantifications, the acceptor cells were divided into three categories; cells with no detectable LDs, cells with a low number of LDs (1–3 LDs/cell) and cells with a high number of LDs (> 3 LDs/cell). Comparison between the two set-ups did not show a significant difference, indicating similar transmission of LDs from control donors and Aβ donors (Fig. 3f, Fig. S5).

To investigate whether LDs, in addition to TNT transfer, could be transported from one cell to another via secretion, we added conditioned medium from donor astrocytes to acceptor astrocytes (Fig. 3g). Analysis after 6 h, revealed LipidTOX positive LDs in the acceptor cells, both when the donors were Aβ-exposed astrocytes (Fig. 3h) and control astrocytes (Fig. 3i).

Taken together, our results show that LDs are transferred between astrocytes via both TNTs and secretion.

### Acute Aβ pathology causes LD accumulation and fatty acid changes in astrocytes

**Fig. 4:**
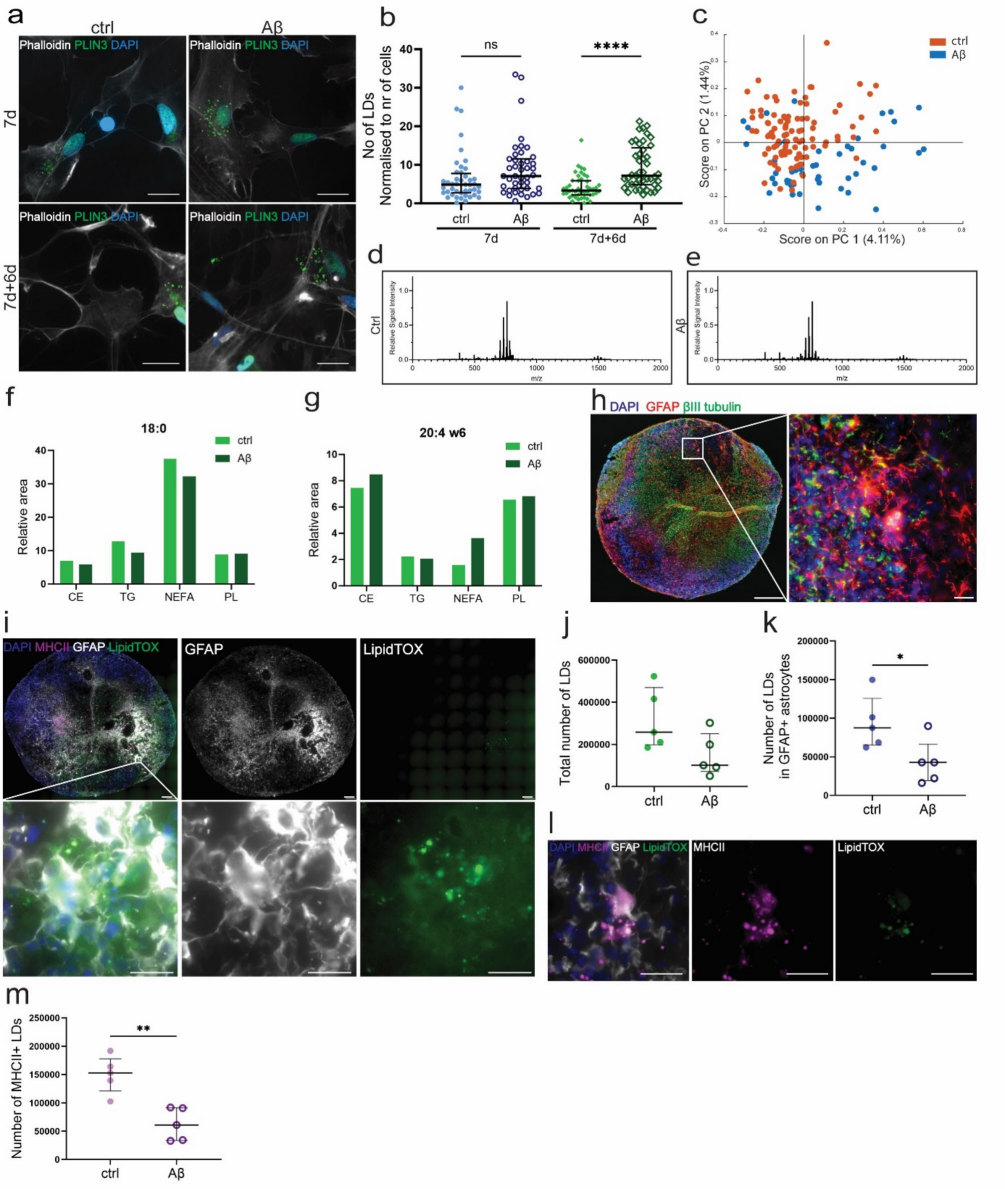
Aβ exposure affects LD metabolism in human astrocytes. PLIN3-ICC of Aβ-exposed astrocytes (**a**), showed an increase in the number of LDs, compared to control astrocytes (**b**). ToF-SIMS showed no differences in total lipid composition between the groups (**c**). Lipidomics confirmed the unchanged lipid profiles between controls (**d**) and Aβ accumulating astrocytes (**e**). However, fatty acid profiling demonstrated that fatty acids 18:0, and 20:4 w6 had changed profiles in Aβ exposed astrocytes (**f**-**g**). ICC of cortical organoids (**h**) exposed to Aβ aggregates demonstrated LDs within the organoid astrocytes (**i**). Quantification of the total number of LDs (**j**) and astrocytic LDs (**k**), revealed a decrease in Aβ-exposed organoids compared to control organoids. Moreover, there was a significant decrease of MHCII + LDs (**l**) in Aβ exposed organoids (**m**). Scale bar (**a**): 20 μm; (zoomed out **h**-**i**): 200 μm; (zoomed in **h**-**i**, **l**): 20 μm. For the statistical analysis of (**b**) the N number is 3, while it was 1 or (**f**) and (**g**) and 5 for (**j**), (**k**) and (**m**). All the data is shown as scatter plots with mean and standard deviation. The level of significance for all the graphs is: * = *P* < 0.05, ** = *P* < 0.01, and *** = *P* < 0.001

Recent studies have shown that the *APOE* genotype (which is linked to the risk to develop AD) can affect lipid content and LD accumulation in glial cells [36–38]. Hence, we next explored the impact of Aβ pathology on LD accumulation, as well as the overall lipid profile in human astrocytes (Fig. S6a). Quantification of PLIN3-ICC (Fig. 4a, Fig. S6b) showed a significant increase of LDs in Aβ-treated astrocytes, compared to untreated controls, at 7d + 6d (Fig. 4b). The total area of all LDs per cell was also significantly increased (Fig. S6c), but the average size of each individual LD remained unchanged (Fig. S6d). Hence, in contrast to our analysis of human brain sections, acute Aβ-exposure stimulated LD accumulation in astrocytes.

To find out if the increase in LDs coincided with an overall change in cellular lipid composition, we performed ToF-SIMS imaging and MALDI-TOF MS on control and Aβ-exposed astrocytes at 7d + 6d. Interestingly, no significant difference between the groups was detected in the ToF-SIMS score plots (Fig. 4c, Fig. S7a-b) or in the lipid profiles from the MALDI TOF-MS (Fig. 4d-e), indicating an intracellular lipid re-distribution, rather than an overall change in lipid composition.

To further study lipid alterations in astrocytes with Aβ pathology, we performed fatty acid profiling at 7d + 6d, focusing on the fractions: cholesteryl esters (CE), triacylglycerols (TG), non-esterified fatty acids (NEFA), and phospholipids (PL) (Fig. S7c). In line with the ToF-SIMS data, the overall fatty acid composition was rather stable. However, we detected a few specific alterations of high interest. Stearic acid (18:0), known to have neuroprotective effects [39, 40], and stearic acid containing lipid species were decreased in Aβ-exposed astrocytes fractions, except as part of PL (Fig. 4f). Concordantly, the levels of fatty acid elongase Elovl6, estimated as the product-to-precursor ratio (18:0/16:0), was decreased in both CE and TG (Fig. S7d). The levels of the fatty acid elongases Elovl 1, 3 and/or 7 were increased in Aβ exposed astrocytes, as estimated based on the product-to-precursor ratio (20:0/18:0) (Fig. S7e). Arachidonic acid (20:4 w6), known to have a role in neuroinflammation [41], was increased in Aβ-exposed astrocytes, in all fractions except TG (Fig. 4g). Increased levels of fatty acid desaturases (delta-5, delta-6 and delta-9) were evident across fractions, especially for TG and NEFA (Fig. S7f-i). Taken together, our data suggest that acute Aβ-pathology affects LD metabolism and specific fatty acids, but not the overall lipid content in astrocytes.

### Chronic Aβ pathology causes a drop in astrocytic LDs in cortical organoids

We have previously shown that Aβ accumulation severely impairs the mitochondria in human astrocytes, causing a metabolic shift to peroxisomal-based fatty acid β-oxidation and glycolysis [35]. Hence, we were interested to study how long-term Aβ pathology affects astrocytic LDs. For this purpose, cortical organoids, containing GFAP + astrocytes and βIII tubulin + neurons (Fig. 4h), were exposed to soluble Aβ aggregates and thereafter maintained in culture for 10 weeks (Fig. S8a). Using ICC, we confirmed the presence of LDs inside GFAP + astrocytes (Fig. 4i). In line with our analysis of tissue sections, we found that the number (Fig. 4j) and total area (Fig. S8b) of LDs were decreased in organoids exposed to Aβ, compared to control organoids. However, the average size of the LDs remained unchanged (Fig. S8c). Quantification of LDs in GFAP + astrocytes specifically, showed a decrease of LDs in Aβ-exposed organoids compared to controls (Fig. 4k). These results suggest that astrocytes subjected to long-term Aβ pathology are not able to keep their LD homeostasis but rather forced to use the LDs as an energy source, which is in line with our analysis of human brain material.

Moreover, quantification of MHCII + LDs in the organoids (Fig. 4l) similarly showed a significant decrease in the Aβ exposed cultures compared to control cultures (Fig. 4m).

## Discussion

Growing evidence indicates a central role of astrocytes in immune regulation in the brain [42, 43], including interaction with T cells and antigen presentation [21, 44]. However, the mechanisms behind the astrocytes’ immune regulatory functions are still unclear. T cells are integral components of the adaptive immune response. Although they are not frequently detected in the healthy brain parenchyma, T cells have been shown to enter the nervous system in significant proportions in various neurological conditions, including AD [45, 46]. The identification of circulating Aβ-reactive T cells has prompted discussions regarding their potential involvement in AD pathology [47], but their interplay with the stationary brain cells remains unclear. Here, we show that CD4 + T cells are in direct contact with GFAP + astrocytes in the AD brain. Interestingly, the astrocyte-connecting T cells display a high LD load, indicating that LDs may play a role in the T cell-astrocyte cross talk.

In AD mice, LD accumulation has been demonstrated to precede the development amyloid plaques [48]. Moreover, LDs have been suggested to accumulate in the AD brain [18, 36, 37]. However, there are several populations of LDs, which may be involved in different processes. To elucidate the impact of LDs in pathological conditions, it is important to distinguish between the LD subclasses, using specific markers. In this study, we show that cultured human astrocytes accumulate PLIN3 + LDs in the acute phase of Aβ pathology. However, in the late-stage AD brain or in cortical organoids with long-term AD pathology there is instead a significant decrease in the number of PLIN3 + LDs, indicating a diverse metabolic dysregulation of this specific LD population over time. In the brain, astrocytes are sites of β-oxidation and when needed they use fatty acids to support mitochondria in their citric acid cycle ATP production [49]. We have previously shown that Aβ accumulation profoundly affects human astrocytes and changes their entire energy metabolism. A high Aβ load is clearly stressful for the astrocytes and associated with an increased energy demand. On top of that, Aβ accumulation affects the mitochondrial dynamics and functionality. However, by shifting their energy production to peroxisomal fatty acid β-oxidation, the astrocytes can maintain functionality and keep constant ATP levels [35]. Hence, the most likely explanation for the later drop in PLIN3 + LDs, as we noted in this study, is that the LDs are utilized by the diseased astrocytes as an energy source when the mitochondria are not able to produce ATP on their own.

In immune cells, LDs are well-established structural markers of ongoing inflammation [6, 50]. Moreover, lipid synthesis and oxidation are known to be vital for survival and differentiation of several CD4 + T cell subclasses [51, 52]. Interestingly, we show that MHCII is situated in the core of astrocytic PLIN3 + LDs, both in the human brain, in cultured astrocytes and in cortical organoids. It is possible that astrocytes not only play a role in antigen presentation, but also contribute to energy homeostasis of the brain-infiltrating T cells. Our data indicate that transportation of LDs between cells are not only a way for the cells to help their neighbors to cope with a high energy demand, but is also directly involved in the inflammatory cross-talk. Our hypothesis is that LDs loaded with MHCII molecules can constitute an “antigen presentation kit”, containing both crucial lipids and antigen presentation molecules that when sent to recipient cells can speed up the T-cell activation. However, it is not all LDs that contain MHCII, indicating that a subclass of LDs plays a key role in antigen presentation. While 2D cultures enable detailed studies of astrocytic LDs and MHCII, the use of 3D organoid cultures constitutes an important intermediate step between the 2D cultures and the human brain. In this study, quantifications of the number of MHCII + LDs, showed a decrease following Aβ exposure in both human astrocytes and organoids, indicating that loading MHCII within LDs is a normal process that occurs in healthy, rather than diseased astrocytes. When LD accumulation was stimulated by exposing the astrocytes to the unsaturated fatty acid OA, there was an increase in both the percentage of MHCII + LDs and total MHCII expression, suggesting an active role of LDs in MHCII antigen presentation.

Exogenous antigens that are ingested by antigen-presenting cells through endocytosis are normally entering the endo-lysosomal pathway, where they are fragmented to 12–24 amino acids. The fragments are then presented to MHC-II, which is transported from the Golgi apparatus to the late endosomes where they bind to each other. The antigen-MHC-II complex is next transported to the cell surface, where it is presented to the CD4 + T helper cells [53]. Interestingly, MHCII positive LDs do not co-localize with lysosomal markers, suggesting that the packing of MHCII into LDs takes place outside of the traditional antigen presentation pathway [32]. To investigate this further, we sought to clarify if MHCII had been processed in the endo-lysosomal pathway at all. An invariant chain (Ii) is found on MHCII before being processed in the lysosomal compartment, where it is cleaved to CLIP and CD74 [32, 54]. Staining for CD74 showed that its pattern was very different in astrocytes with a “normal” MCHII expression (where CD74 seemed to be present in the same compartments as MHCII) and in astrocytes where MHCII was situated inside LDs (where CD74 did not at all co-localize with MHCII). Moreover, staining with the conformation specific clone L432 antibody [55], confirmed that MHCII molecules packaged into LDs are functional. Taken together, these results indicate that the MHCII found in LDs has been processed in the lysosome and is available for antigen presentation.

Notably, we demonstrate that human astrocytes load MHCII complexes within PLIN3 + LDs and forward the “MHCII-lipid packages” to neighboring cells via tunneling nanotubes. Tunneling nanotubes are a well-known route of cell-to-cell communication that can also be involved in pathological processes, such as spreading of toxic protein aggregates [56]. Our previous studies have shown that astrocytes transfer aggregated proteins as well as mitochondria within TNTs [33–35, 57, 58] and we hereby add MHCII-containing LDs to this list. Lipid droplets have been shown to play a role in cross-presentation and T cell activation in dendritic cells [5–8], but a direct role of LDs in MHCII transportation within and between cells have not been described previously. Astrocytes are extremely secretory cells and we were therefore interested in investigating if they also use exocytosis to transfer LDs. Indeed, we found LDs to be transported between astrocytes via conditioned medium. It is known that healthy astrocytes can transfer mitochondria to rescue stressed neighbors [34]. However, based on our donor-acceptor studies, we draw the conclusion that Aβ-pathology does not affect astrocytic LD transfer, at least not in the acute setting, and that this is not primarily a way for the astrocytes to maintain lipid homeostasis under stress.

Astrocytes play a critical role in lipid metabolism and the prevention of neuronal lipotoxicity, by removing and storing excess fatty acids [2, 59]. Our previous studies show that astrocytes accumulate Aβ, leading to cellular stress and metabolic changes [35, 60]. Other studies indicate that the sporadic AD risk factor ApoE4, causes metabolic and lipid dysregulation in glial cells [36–38]. Hence, we sought to investigate if Aβ pathology would affect the astrocytic lipid metabolism. Analysis of the overall lipid content, using ToF-SIMS and MALDI-TOF MS, did not show significant changes between the groups, suggesting reorganization of lipids rather than a difference in content. To further investigate the lipid content, we performed fatty acid profiling. Although the spectra were not completely altered by Aβ exposure, specific fatty acids showed differences. Interestingly the neuroprotective fatty acid stearic acid [39, 40] was decreased in Aβ accumulating astrocytes, while the cytokine precursor arachidonic acid [61] was increased. The differences were observed in the neutral lipids fractions CE and TG of the samples, which most likely reflect the LDs. It is well documented that LDs can engage in various inflammatory signaling pathways by transforming their constituent lipids into active lipid mediators. For example, in leukocytes, LDs have been assigned for their unique capacity to generate eicosanoids from arachidonic acid [62]. Many key enzymes involved in the biosynthesis of eicosanoids, including lipoxygenase and cyclooxygenase are actually specifically located within LDs in activated immune cells [62].

Increasing evidence highlights the diverse and dynamic role of LDs in various cell types [50, 63]. Our study extends the field by investigating the role of LDs in astrocyte-mediated neuroinflammation in the context of AD. Importantly, we describe a novel function of PLIN3 + LDs in harboring and transporting functional MHCII complexes that may constitute a distinct antigen presentation pathway. Our findings contribute to basic knowledge about the neuroinflammatory machinery that may be central for many brain conditions, including other neurodegenerative diseases and brain tumors.

## Electronic supplementary material

Below is the link to the electronic supplementary material.

Supplementary Material 1

Supplementary Material 2: Movie S1. Time lapse movie of human astrocytes, labelled with BODIPY493/505 (red), show a sender astrocyte (S) that pack and transfer LDs (Arrow) via TNTs to a recipient astrocyte (R). Fig. 3b presents snapshots from the movie

## Data Availability

No datasets were generated or analysed during the current study.

## Abbreviations

Aβ: Amyloid beta
AD: Alzheimer’s disease
CEs: Cholesterol esters
FA: Fatty acid
hiPSCs: Human induced pluripotent stem cells
IHC: Immunohistochemistry
ICC: Immunocytochemistry
LDs: Lipid droplets
MALDI-TOF MS: Matrix-Assisted Laser Desorption/Ionization Time-of-Flight Mass Spectrometry
MHC-II: Major Histocompatibility Complex II
NEFAs: Non-esterified fatty acids
NK cells: Natural Killer cells
OA: Oleic acid
PCA: Principal Component Analysis
PFA: Paraformaldehyde
PLA: Proximity Ligation Assay
PLIN: Perilipin
PLs: Phospholipids
TAGs: Triacylglycerols
TEM: Transmission Electron Microscopy
TNTs: Tunneling Nanotubes
ToF-SIMS: Time-of-Flight Secondary Ion Mass Spectrometry
