# Supplementary material for: Astrocytic lipid droplets contain MHCII and may act as cogs in the antigen presentation machinery
Source: J Neuroinflammation. 2025 Apr 24;22:117. doi: 10.1186/s12974-025-03452-0 (PMC12023685; doi:10.1186/s12974-025-03452-0)
Supplement: Supplementary file 1 — Supplementary Material 1 [file 12974_2025_3452_MOESM1_ESM.docx]

## **Supplementary Figures and Tables**


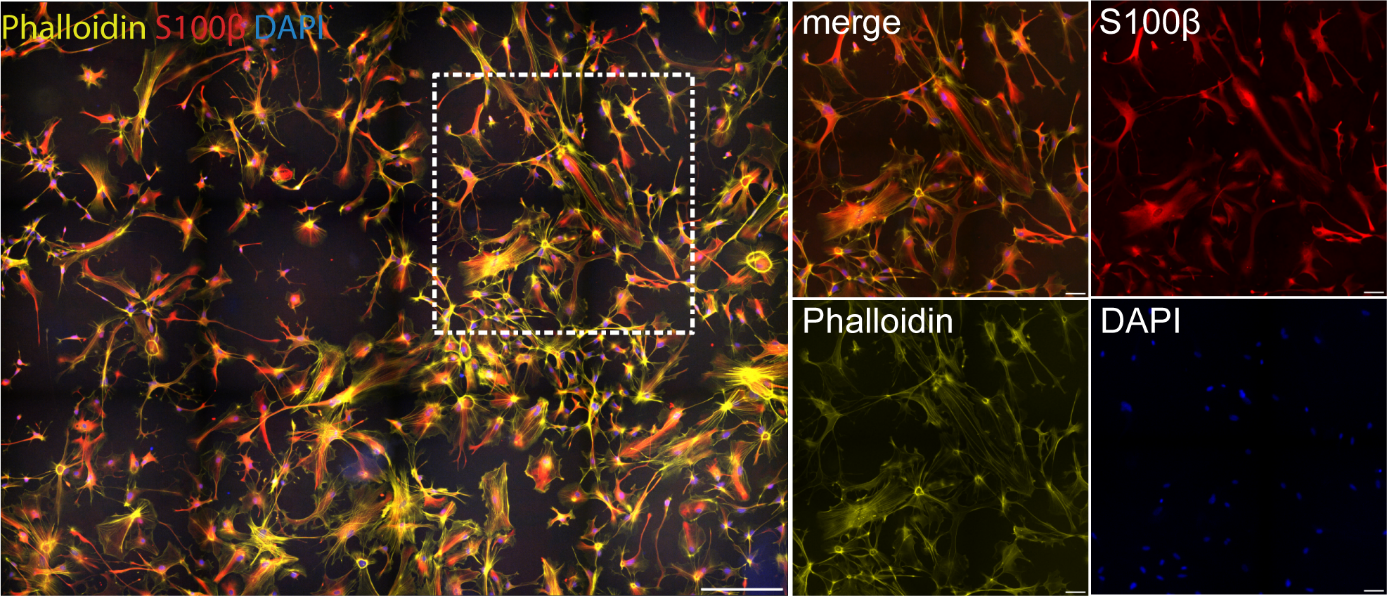

**Figure S1. Astrocytic composition of the culture model.** ICC staining with the astrocytic marker S100β and the actin dye phalloidin, showing a close to 100% astrocytic representation in the cultures. Scale bar: 250 µm, (zoomed in) 50 µm.


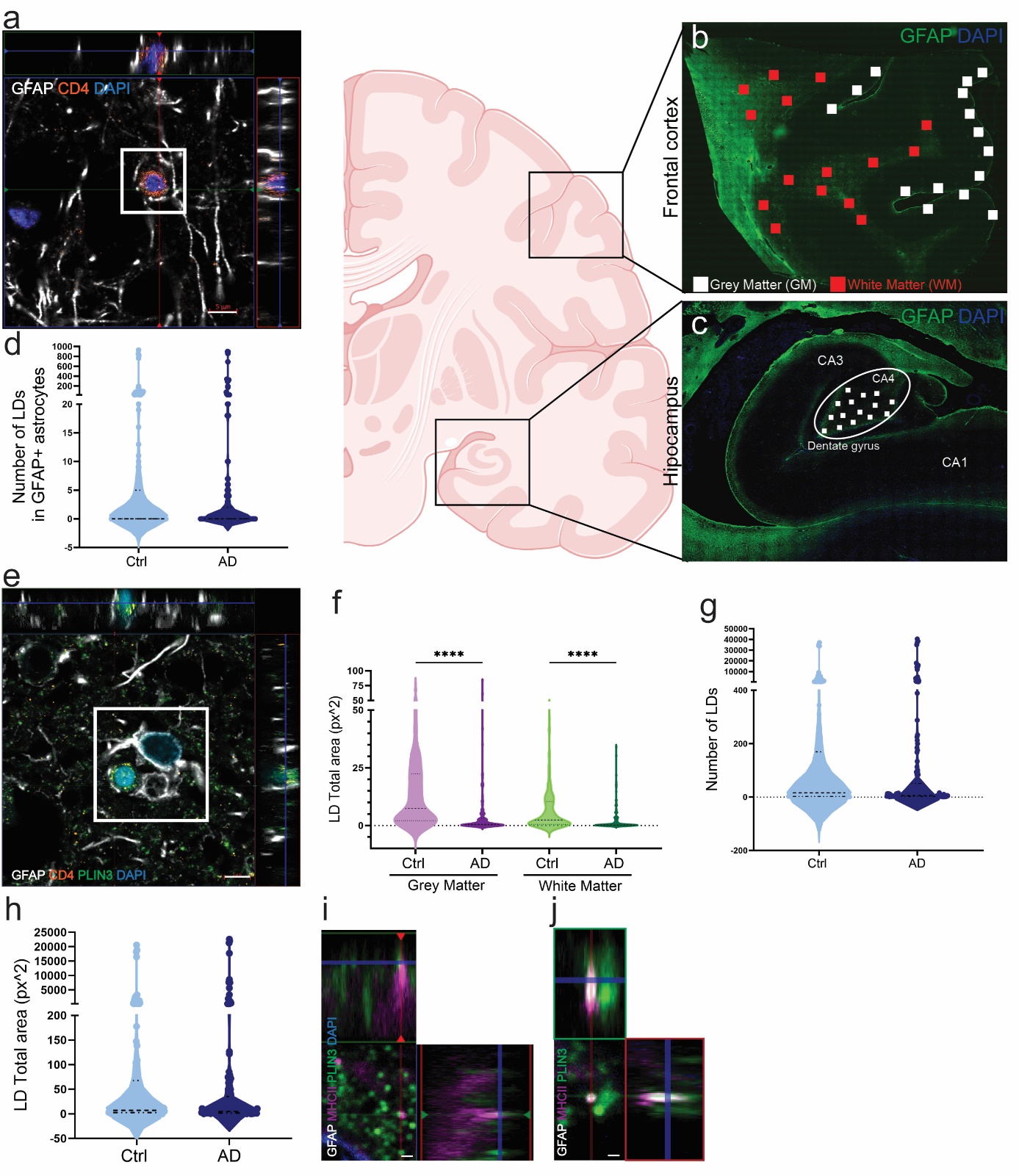


**Figure S2. Cortical and hippocampal astrocytes in the AD brain are positive for MHCII and PLIN3.** Overview of IHC stainings of cortical AD brain sections, using GFAP and CD4 antibodies (a, Fig. 1a). Schematic representation of the captured images of frontal cortex (b, in grey matter (white squares), and in white matter (red squares)), and of hippocampus (c). Quantification of PLIN3+LDs in GFAP+ astrocytes in the hippocampus showed no significant difference (d). Overview of IHC stainings of cortical AD brain sections PLIN3, CD4 and GFAP antibodies (e, Fig. 1e), showing astrocytes in contact with lipid-laden CD4+ T cells. PLIN3+LD area showed significant decrease in AD cortex compared to control cortex in both grey and white matter (f), while there was no significant difference in the total number and area of PLIN3+LDs in hippocampus (g-h). PLIN3+LDs co-localized with MHCII in hippocampus (i-j, Fig 1h). Scale bar: (a-b) 5 µm; (i-j): 1 µm.

*
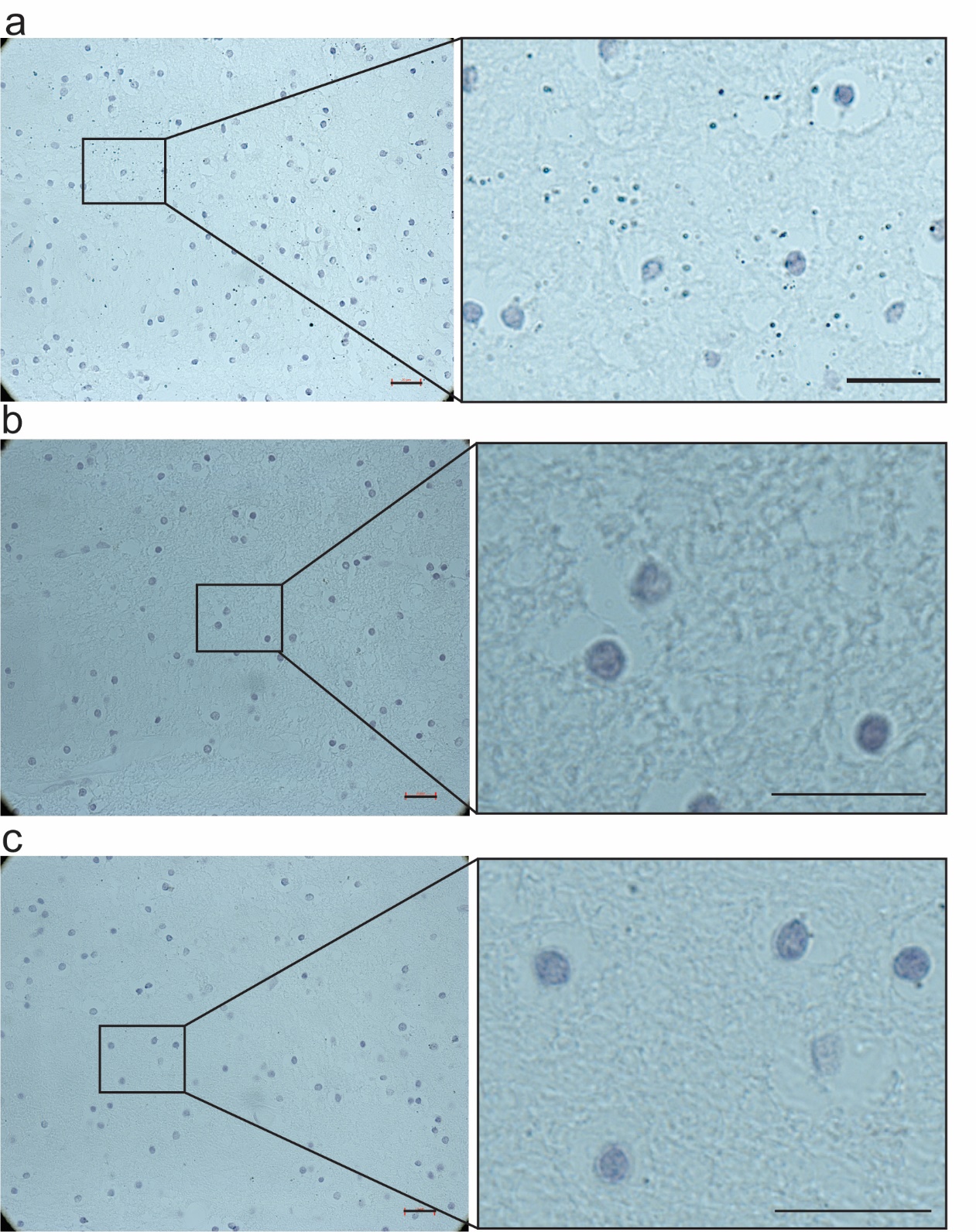
*

**Figure S3. MHCII and PLIN3 interact and form complexes in the AD patient.** Overview of PLA analysis of cortical AD brain sections, using antibodies against PLIN3 and MHCII, showing positive signal throughout (a, Fig. 1j)). The negative PLA controls, performed without the secondary antibody against MHCII (b-c), showed very little background signal. Scale bar: 20 µm.

**
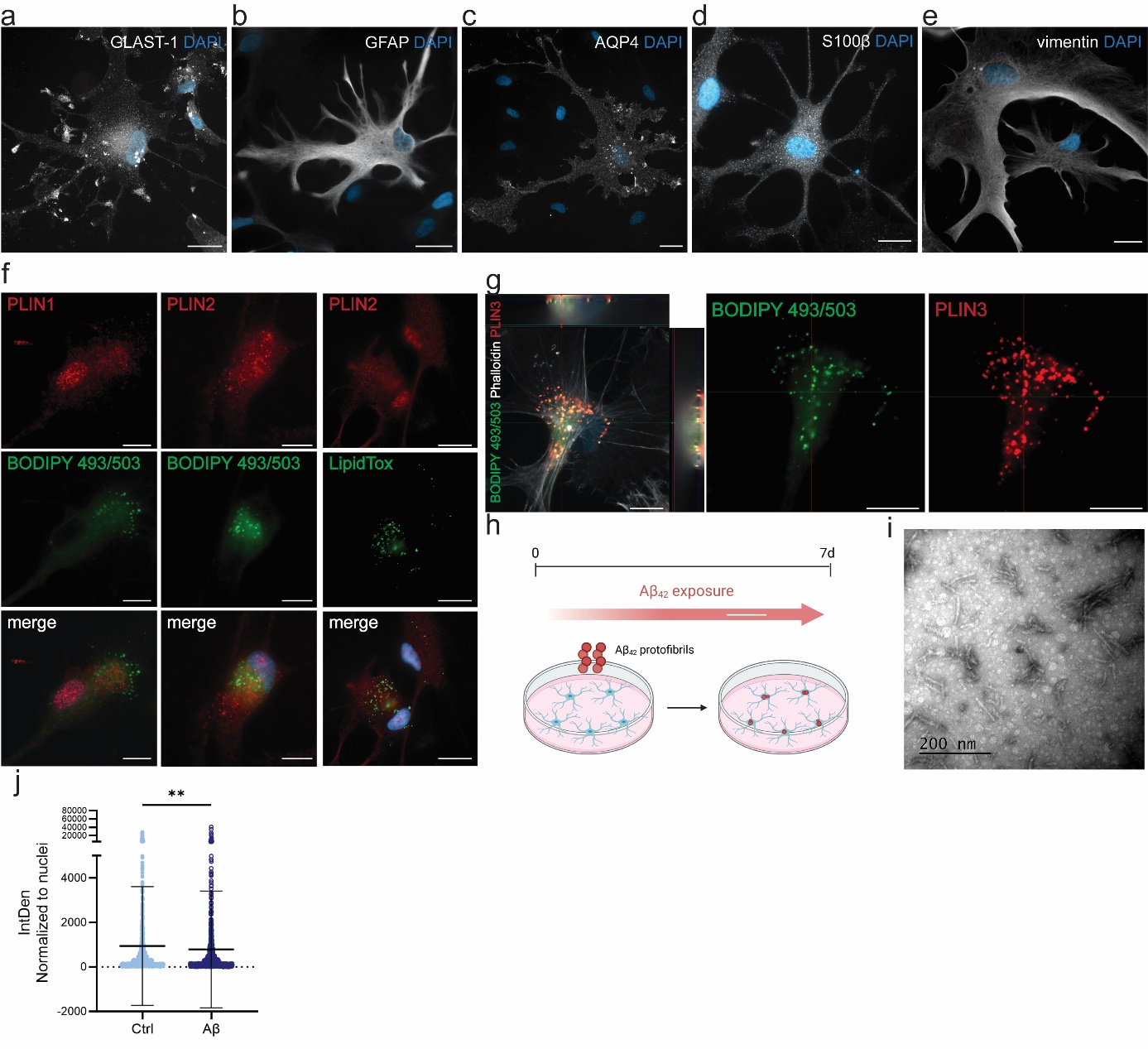
**

**Figure S4. hiPSC-derived astrocytes, expressing multiple astrocytic markers, contain PLIN3 coated LDs.** ICC demonstrated that hiPSC-derived astrocytes were positive for GLAST-1 (a), GFAP (b), AQP4 (c), S100β (d), and vimentin (e). hiPSC-derived astrocytes stained with BODIPY493/503 or LipidTOX showed no co-localization of LDS with PLIN1 or PLIN2 (f), but PLIN3 clearly coating the BODIPY 493/503 positive LDs (g). Schematic illustration of experimental setup (h). TEM of soluble Aβ aggregates (i). Quantification of MHCII+ LDs showed a significant decrease in total MHCII in Aβ accumulating astrocytes (j).Scale bar: 20 µm.


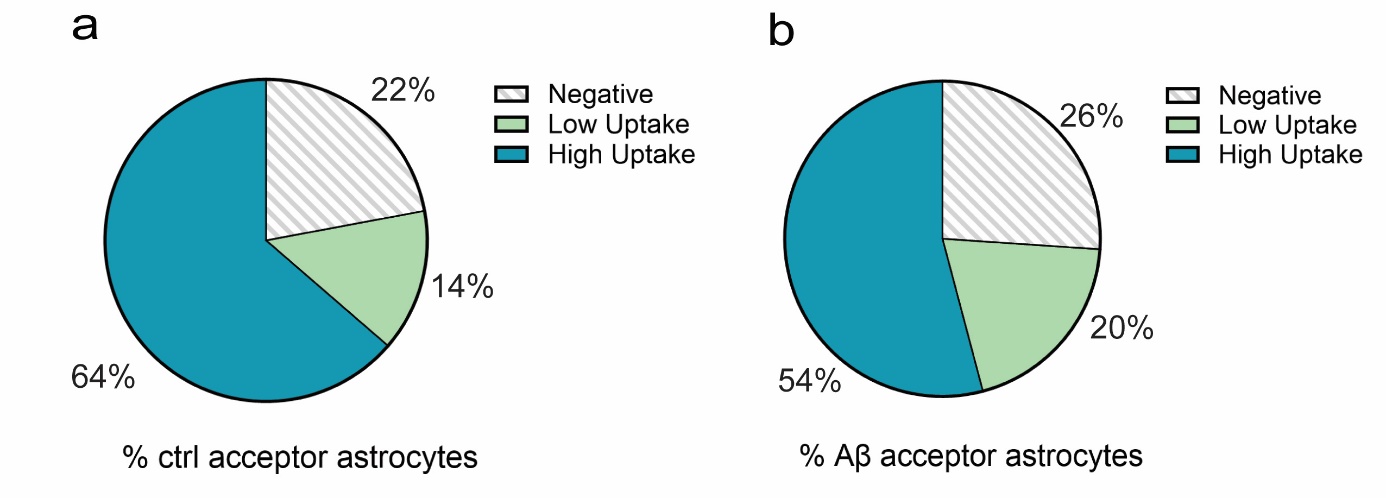


**Figure S5. Astrocytes transfer LDs.** Quantification of acceptor astrocytes with no detectable LDs (negative), low number of LDs (1-3 LDs/cell), and high number of LDs (>3 LDs/cell) demonstrated that the majority of the acceptor cells had a high number of LDs in both a set-ups; (a) donor cells had Aβ pathology and the acceptor cells were untreated or (b) the donor cells were untreated and acceptor cells had Aβ pathology.


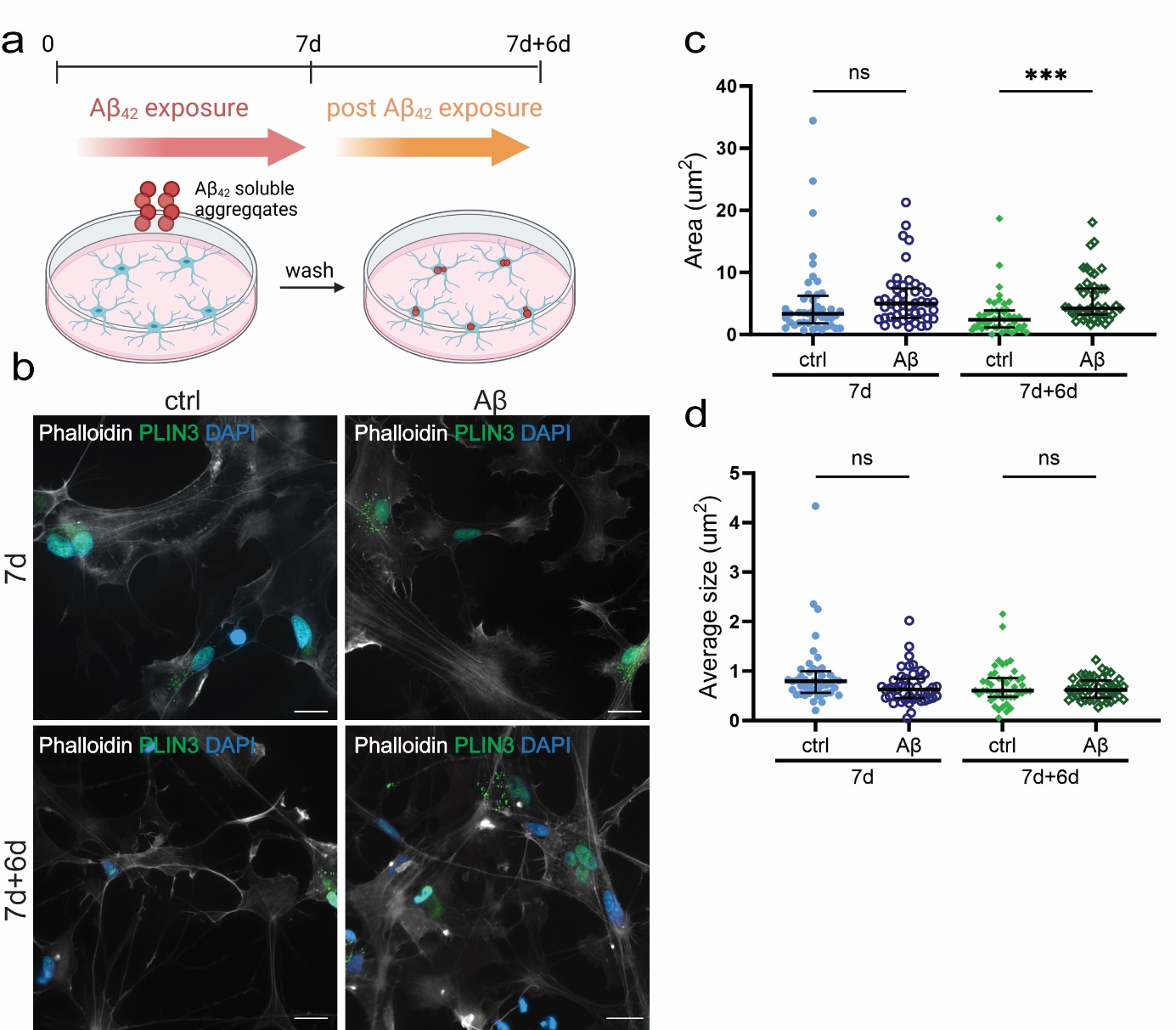


**Figure S6. Increased number of LDs in Aβ accumulating astrocytes.** Schematic illustration of experimental setup (a). Overview pictures of control and Aβ exposed astrocytes at 7d and 7d+6d, stained for Phalloidin, PLIN3 and DAPI (b, Fig 4a). Quantification of LDs showed an increase in their area in Aβ exposed astrocytes compared to control astrocytes at 7d+6d (c), while the LD average size remained unchanged between the groups (d). Scale bar: 20 µm.


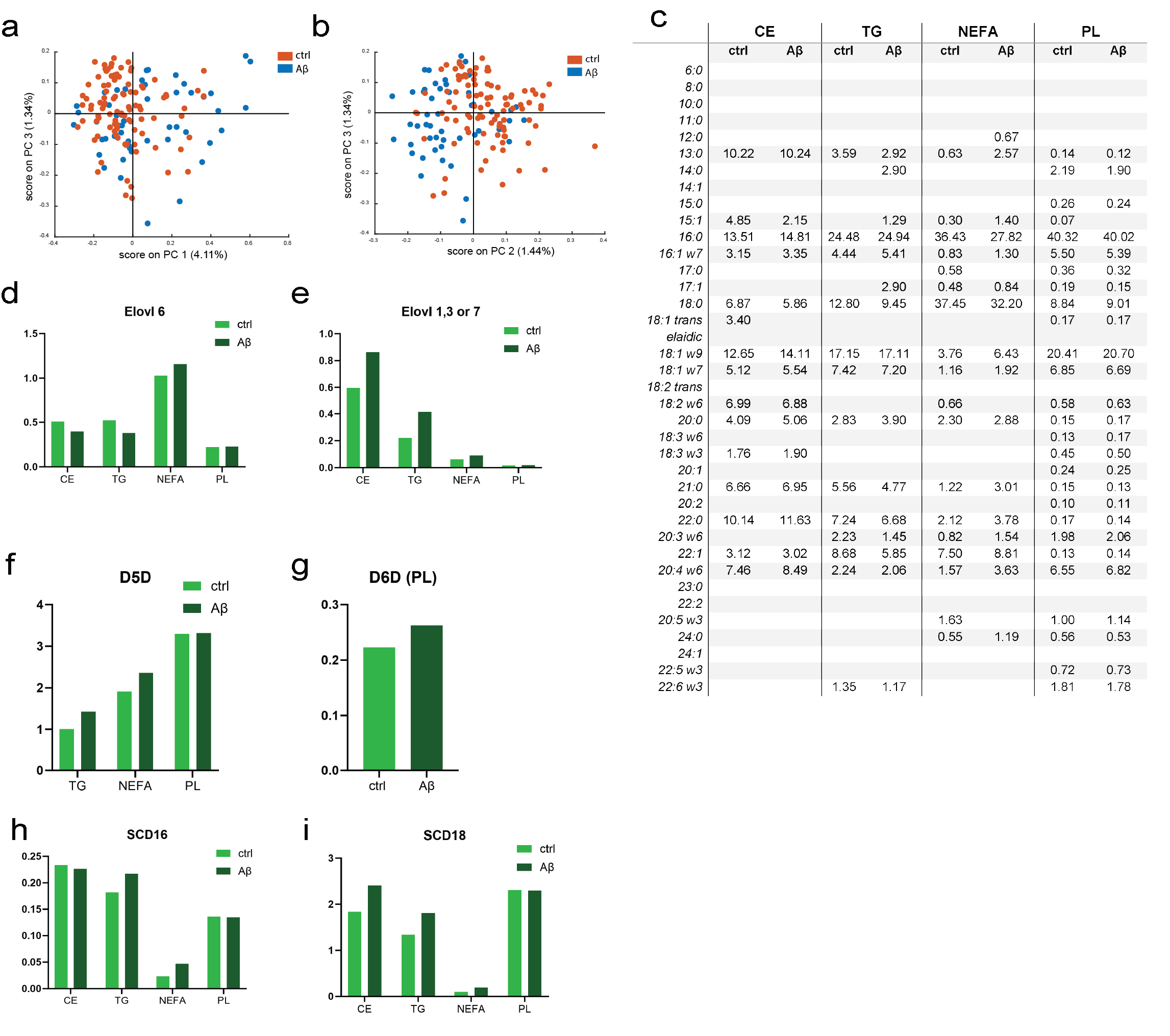


**Figure S7. Astrocytes exposed to Aβ show no changes in total lipid composition.** Score plot of PC1 and 3 (a), and PC2 and 3 (b), in negative ion mode, m/z 200-1600. Fatty acid analysis showed a distinct profile of astrocytes in four different fractions: cholesteryl esters (CE), triacylglycerols (TG), non-esterified fatty acids (NEFA), and phospholipids (PL) (shown as relative area of the whole fraction) (c). The activity of elongases and desaturases (estimated as product-to-precursor ratios) were also assessed. Elongase Elovl6 showed to be decreased in CE and TG but increased in NEFA in Aβ-exposed astrocytes (d), while Elovl 1, 3 or 7 was decreased in the controls in all fractions (e). Delta-5 desaturase (D5D) showed decreased levels in TG and NEFA in controls, but not in PL (f), while delta-6 desaturase (D6D) showed increased levels in Aβ-exposed astrocytes (g). Delta-9 desaturase was analyzed by looking at stearoyl-CoA desaturase (SCD)16 which showed increased levels in Aβ accumulating astrocytes in TG and NEFA (h), and SCD18, that showed increased levels in all fractions except PL in Aβ-exposed astrocytes (i).


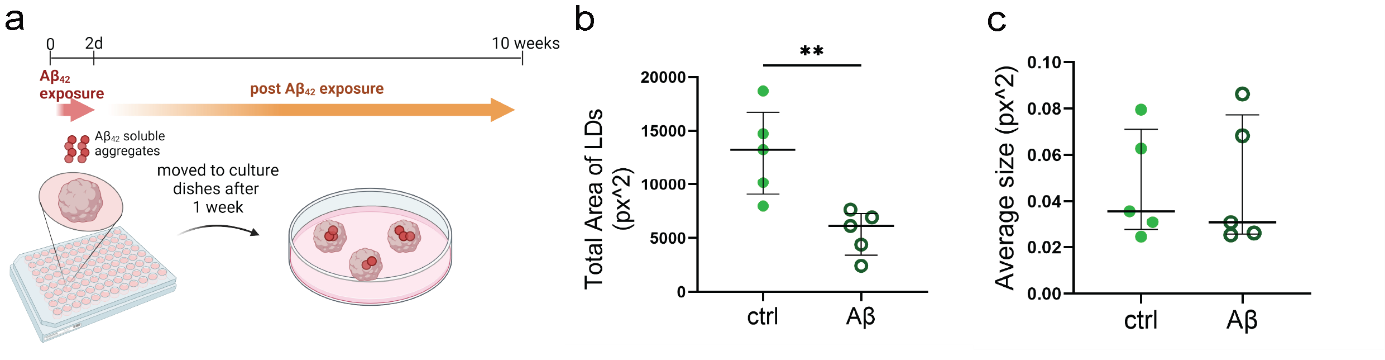


**Figure S8. Aβ pathology causes decrease in PLIN3+ LDs in cortical organoids.** Schematic illustration of experimental setup for cortical organoid experiments (a). Quantification of LDs showed a decrease in total LD area in Aβ exposed organoids compared to control organoids (b), while the LD average size remained unchanged (c).

/*

**QUANTIFICATION OF PLIN3 LD in astrocytes**

The threshold (min and max) and pixel size (min and max) were set manually for each experiment and subsequently applied to all images.

/*

**// Counting the number of astrocytic PLIN3 LD in human brain tissue**

//Automation

inputfolder = "C:\\Input\\";

outputfolder = "C:\\Output\\";

inputfilelist = getFileList(inputfolder);

for(k=0;k<lengthOf(inputfilelist);k=k+2) {

roiManager("Reset");

open(inputfile + inputfilelist[k]);

print(k);

C2location=lastIndexOf(inputfilelist[k],"c2");

print(C2location);

name=inputfilelist[k];

newname=replace(name, "c2", "c3");

print(name);

open(inputfile + newname);

selectImage(name);

//Measurement outline channel (c2) GFAP

run("Set Scale...", "distance=1 known=0.103 pixel=1 unit=micrometer");

run("Subtract Background...", "rolling=50");

run("8-bit");

call("ij.plugin.frame.ThresholdAdjuster.setMode", "Red");

setThreshold(15, 255);

setOption("BlackBackground", true);

run("Convert to Mask");

run("Fill Holes");

run("Sharpen");

run("Despeckle");

run("Analyze Particles...", "size=30-100000 pixel circularity=0-1.00 show=Masks");

run("Invert");

run("Create Selection");

roiManager("Add");

//Measurement channel of interest (c3) PLIN3

selectImage(newname);

run("Set Scale...", "distance=1 known=0.103 pixel=1 unit=micrometer");

run("Subtract Background...", "rolling=50");

run("8-bit");

roiManager("Select", 0);

run("Clear Outside");

roiManager("select", 0);

setThreshold(35, 255);

setOption("BlackBackground", true);

run("Convert to Mask");

run("Smooth");

run("Make Binary");

run("Watershed");

run("Analyze Particles...", "size=15-400 pixel circularity=0.10-1.00 show=Masks display summarize add show=Masks");

a=getTitle();

}

**Table S1.** ImageJ macro for quantification of astrocytic PLIN3+LDs in human brain tissue.

/*

**QUANTIFICATION OF PLIN3 LD**

The threshold (min and max) and pixel size (min and max) were set manually for each experiment and subsequently applied to all images.

/*

**// Counting the number of PLIN3 LD in human brain tissue**

//Automation

inputfolder = "C:\\Input\\";

outputfolder = "C:\\Output\\";

inputfilelist = getFileList(inputfolder);

for(k=0;k<lengthOf(inputfilelist);k=k+1) {

filename=inputfilelist[k];

open(inputfolder + filename);

print(k);

print(filename);

selectImage(filename);

//Measurement

run("Set Scale...", "distance=1 known=0.103 pixel=1 unit=micrometer");

run("Subtract Background...", "rolling=50");

run("8-bit");

setThreshold(35, 255);

setOption("BlackBackground", true);

run("Convert to Mask");

run("Smooth");

run("Make Binary");

run("Watershed");

run("Analyze Particles...", "size=15-400 pixel circularity=0.10-1.00 show=Masks display summarize add show=Masks");

}

**Table S2.** ImageJ macro for quantification of total PLIN3+LDs in human brain tissue.

/*

**QUANTIFICATION OF MHCII+LD**

The threshold (min and max) and pixel size (min and max) were set manually for each experiment and subsequently applied to all images.

/*

**// Counting the number of MHCII+LD**

//Automation

if (nImages>0) run("Close All"); // if there are 1 or more images - close all

if (isOpen("Results")) { // close results window, if open

selectWindow("Results");

close("Results");

}

print("\\Clear"); // empty log window

roiManager("reset"); // empty ROI manager

run("Options...", "iterations=1 count=1 black edm=32-bit"); // set Binary Options

//File opening

setBatchMode(1);

openfolder1= getDirectory("Choose your Outline channel");

openfolder2= getDirectory("Choose your channel of intrest");

ListArray1 = getFileList(openfolder1);

ListArray2 = getFileList(openfolder2);

print("Number of processed files",ListArray1.length);

print("Number of processed files",ListArray2.length);

//Measurement outline channel PLIN3

for (i = 0; i < ListArray1.length; i++) {

filename1=ListArray1[i];

open(openfolder1+filename1);

run("8-bit");

run("Subtract Background...", "rolling=50");

setOption("ScaleConversions", true);

run("Set Scale...", "distance=99 known=10 unit=um");

setThreshold(55, 255);

run("Convert to Mask");

run("Watershed");

run("Analyze Particles...", "size=0.01-10 circularity=0.10-1.00 show=Masks display clear summarize add");

Roicount= roiManager("count");

if (Roicount > 0) {

roiManager("Select", 0);

roiManager("Add");

print("Total number of LDs", Roicount);

//Measurement channel of interest MHCII

filename2=ListArray2[i];

open(openfolder2+filename2);

run("8-bit");

run("Subtract Background...", "rolling=50");

setOption("ScaleConversions", true);

run("Set Scale...", "distance=99 known=10 unit=um");

setThreshold(40, 255);

run("Convert to Mask");

run("Watershed");

Roicount= roiManager("count");

minusonecount = Roicount-1;

for (m = Roicount-1; m >=0; m=m-1) {

roiManager("select",m) ;

run("Measure");

tablepos1 = minusonecount-m;

Meaninten = getResult("Mean");

if (Meaninten == 0) {

roiManager("delete");

}

}

} else {

print("No selection for file " + filename1);

}

newcount=roiManager("count");

print(filename1,"MHC pos LDs",newcount);

close("*");

roiManager("reset");

}

**Table S3.** ImageJ macro for quantification of MHCII+LDs.

/*

**QUANTIFICATION OF PLIN3 LD**

The threshold (min and max) and pixel size (min and max) were set manually for each experiment and subsequently applied to all images.

/*

**// Counting the number of PLIN3 LD**

//Automation

inputfolder = "C:\\Input\\";

outputfolder = "C:\\Output\\";

inputfilelist = getFileList(inputfolder);

for(k=0;k<lengthOf(inputfilelist);k=k+1) {

filename=inputfilelist[k];

open(inputfolder + filename);

print(k);

print(filename);

selectImage(filename);

//Measurement

run("8-bit");

run("Subtract Background...", "rolling=50");

run("Set Scale...", "distance=123 known=20 unit=um");

run("Threshold...");

setThreshold(25, 255);

setOption("BlackBackground", false);

run("Convert to Mask");

run("Set Measurements...", "area mean min integrated area_fraction display redirect=None decimal=1");

run("Analyze Particles...", "size=0.01-10 circularity=0.10-1.00 show=Masks display clear summarize"); }

**Table S4.** ImageJ macro for LD quantification.

**Supplementary Movie**

**Movie S1.** Time lapse movie of human astrocytes, labelled with BODIPY493/505 (red), show a sender astrocyte (S) that pack and transfer LDs (Arrow) via TNTs to a recipient astrocyte (R). Fig. 3b presents snapshots from the movie.
